# Is pregnancy feasible in women with adult granulosa cell tumors, and how should recurrence during pregnancy be managed? A case report and literature review

**DOI:** 10.1016/j.gore.2026.102049

**Published:** 2026-02-17

**Authors:** Moritz Matthaei, B. Gebauer, A. Kunze, W. Schmitt, D. Dimitrova, J. Sehouli

**Affiliations:** a Department of Gynecologic Oncology, Charité University Hospital, Berlin, Germany; b Department of Radiology, Charité University Hospital, Berlin, Germany; c Department of Pathology, Charité University Hospital, Berlin, Germany

**Keywords:** Adult granulosa cell tumor, Pregnancy, Fertility preservation, Recurrence, Case report

## Abstract

**Objective:**

Granulosa cell tumors are rare ovarian malignancies of sex cord-stromal origin. Improved diagnostics and growing clinical awareness have led to increased recognition in young women of reproductive age, emphasizing the need for individualized treatment strategies that balance oncologic safety with fertility preservation. Despite the increasing relevance of fertility preservation in women with granulosa cell tumors, data on pregnancy and the management of disease recurrence during gestation remain extremely limited, with only few cases reported in the literature.This case may serve as a reference for managing similar cases of granulosa cell tumor or its recurrence during pregnancy.

**Case presentation:**

A woman was diagnosed with FIGO IC adult granulosa cell tumor (aGCT) and underwent fertility-sparing surgery followed by adjuvant chemotherapy. The desired pregnancy occurred spontaneously after treatment, but a relapse of the disease was diagnosed during the pregnancy.

**Methods:**

We retrospectively analyzed clinical, histopathological, and medical imaging data of a patient diagnosed with an aGCT that was treated at our institution. Relevant information was collected from the patient’s medical records to illustrate the diagnostic process, treatment, and outcome.

**Results:**

The patient developed two recurrences of an aGCT, one of which occurred during early pregnancy. Surgical management included tumor resection with sigmarectal resection and creation of a descendostomy in the 13th week of gestation. Due to the rarity of such cases, therapeutic planning was based on limited evidence and required an individualized approach. Ultimately, both mother and child remained clinically stable, and the pregnancy proceeded without further complication.

**Conclusion:**

This case illustrates the complexity of managing recurrent aGCT during pregnancy and highlights the importance of individualized, multidisciplinary treatment strategies. Long-term follow-up remains essential due to the risk of late recurrence.

## Highlights

• Rare recurrence of adult granulosa cell tumor during pregnancy.
• Feasibility of pregnancy after fertility-sparing treatment.
• Management strategies for tumor recurrence in gestation.
• Clinical guidance for multidisciplinary decision-making.
• Adds insight for clinicians treating rare ovarian tumors in pregnancy.

## 1 Introduction

Adult-type granulosa cell tumors constitute a rare subset of ovarian neoplasms, accounting for approximately 2–5% of all ovarian malignancies (Ray-Coquard et al., 2014). They are typically characterized by an indolent clinical course but a notable propensity for late recurrence, sometimes decades after the initial diagnosis (Schumer and Cannistra, 2003). Owing to their frequent hormone production − particularly estrogen − patients may present with endocrine symptoms such as abnormal uterine bleeding or endometrial hyperplasia (Schumer and Cannistra, 2003).

Given that many patients are premenopausal at diagnosis, fertility preservation has become an increasingly relevant aspect of clinical management (Ray-Coquard et al., 2014). Several retrospective studies and case series suggest that fertility-sparing surgery may be oncologically acceptable in early-stage disease when combined with adequate staging and close follow-up (Mangili et al., 2013). Nevertheless, the available evidence remains limited due to the rarity of this tumor type, and clear recommendations regarding the safety of pregnancy after treatment are lacking (Ray-Coquard et al., 2014).

Pregnancy after aGCT raises additional clinical challenges. Hormonal changes during gestation—particularly elevations in estrogen, progesterone, and hCG—may theoretically stimulate residual tumor cells (Jamieson and Fuller, 2012). At the same time, monitoring during pregnancy is complicated by physiological alterations in tumor markers and restrictions in imaging modalities. Only a small number of cases describing pregnancy after aGCT or recurrence during pregnancy have been published to date, leaving clinicians with considerable uncertainty when advising affected patients (Zanagnolo et al., 2004).

Here, we report the complex course of a woman with FIGO stage IC aGCT who experienced two recurrences, including one during early pregnancy. This case highlights the diagnostic and therapeutic challenges associated with managing recurrent disease during gestation and underscores the need for individualized, multidisciplinary decision-making.

## 2 Case presentation

In January 2013, the patient presented after a laparoscopic cystectomy on the right adnexa with histological confirmation of an aGCT FIGO stage IC (external) at the Charité Gynaecological Clinic. Taking into account all previous findings as well as the patient's desire for fertility preservation, a fertility-sparing surgery was recommended by unilateral adnexectomy of the affected side, infracolic omentectomy, fractional curettage, and peritoneal biopsies. This procedure was performed laparoscopically in March 2013, with histological examination revealing small tumor manifestations in the mesovarium. With a proliferation marker (MIB-1) growth fraction of 30%, adjuvant chemotherapy with three cycles of paclitaxel and carboplatin was administered.

After the completion of chemotherapy with carboplatin and paclitaxel in July 2013, the patient was in good general condition, with reversible alopecia and only mild sensory disturbances in the left thigh. Despite reduced fertility (AMH 0.8), the patient became pregnant in 2014, with an uneventful pregnancy. Since the diagnosis, the patient has undergone close follow-up care.

In July 2018, a significant increase in tumour markers (Inhibin B) led to the suspicion of a recurrence of the tumour. Imaging demonstrated tumour-specific findings in the pelvis with solid pararectal lesions and close contact to the rectum. In the context of a known history of aGCT and rising inhibin B levels, these findings were considered highly suggestive of tumor recurrence, with definitive confirmation obtained intraoperatively. At this point, the patient was pregnant at 14 weeks gestation. After careful risk assessment, surgery during pregnancy was recommended. The procedure was performed through a lower abdominal transverse laparotomy with tumor resection, partial Douglas deperitonealization, and anterior rectal resection with transanal stapler anastomosis. Postoperatively, no residual tumor was found at the site. The case was discussed in a tumor conference, and different treatment options were presented. At a low proliferation rate (MIB-1 < 5%) an observational approach until the 37th/38th week of pregnancy, followed by delivery via cesarean section and postpartum reevaluation, versus spontaneous delivery with laparoscopic surgery 10–12 weeks post-operation, was discussed in the tumor conference. The proliferation index (Ki-67/MIB-1) was considered as an additional biological parameter in therapeutic decision-making. Although no standardized cut-off values exist for adult granulosa cell tumors, retrospective studies indicate that lower Ki-67 indices are generally associated with a more indolent course, whereas higher values have been described in more aggressive or recurrent disease. For this reason, Ki-67 is frequently used as a supplementary factor when estimating tumor dynamics and supporting individualized treatment decisions, particularly in complex clinical situations such as pregnancy. Based on the tumor board recommendation and the low proliferation index (MIB-1 approximately 5%), an observational management approach during pregnancy was considered appropriate. The options discussed included delivery at 37–38 weeks of gestation, either by cesarean section or spontaneous vaginal delivery, followed by reassessment and possible laparoscopic surgery 10–12 weeks postpartum. Alternatively, systemic treatment options such as chemotherapy or antihormonal therapy with an aromatase inhibitor were considered. Ultimately, in January 2019, a spontaneous delivery occurred. A postpartum MRI showed no evidence of metastatic lymphadenopathy or distant metastasis, and no further surgical intervention was required.

**Figure:**
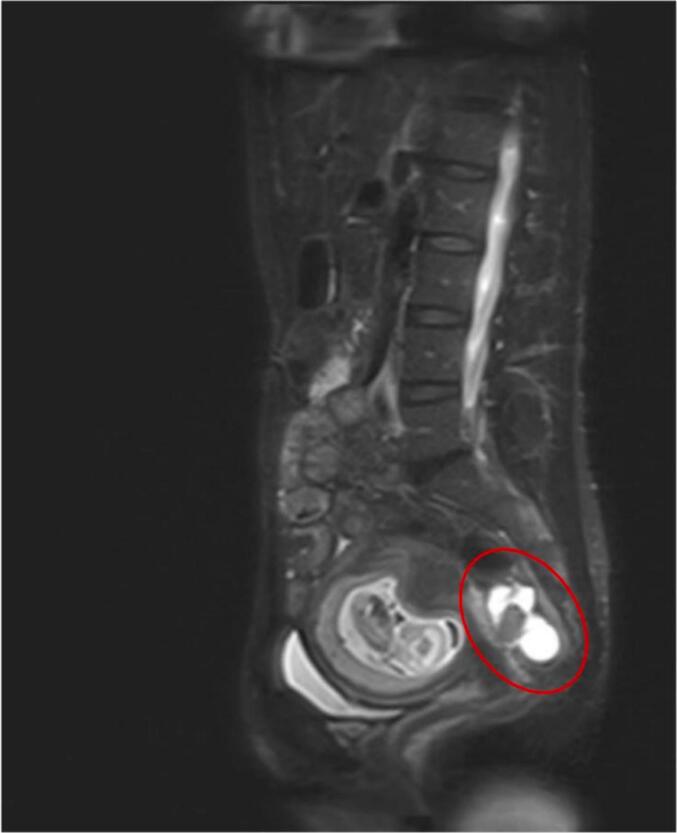

I) July 2018, T2-weighted magnetic resonance imaging of the pelvis demonstrating a pararectal lesion (red circle) with close contact to the rectum, consistent with suspected recurrent adult granulosa cell tumor.

Subsequent follow-up was unremarkable until November 2021, when suspicion of a second tumor recurrence arose. Imaging showed multiple peritoneal nodules, leading to an initial diagnostic laparoscopy, followed by a transverse laparotomy. Tumor resection was performed on the left side of the Douglas pouch, where it adhered to the iliac vessels, ureter, and sigmoid. Additionally, Douglas deperitonealization, uterine deperitonealization of the anterior wall, removal of a nodule from the ventral abdominal wall, a nodule from the cecum, and a sample from the left ovary were carried out. No evidence of peritoneal seeding was observed in the upper abdomen. The site was macroscopically tumor-free, with complete tumor resection. Pathology revealed an adult granulosa cell tumor with a low proliferation index (Ki67 3%). Due to tumor involvement of the left ovary and uterine serosa, a follow-up operation in the form of adnexectomy and supracervical hysterectomy was recommended, aiming to reduce the risk of microscopic tumor spread. In the setting of recurrent disease, removal of the uterine corpus was considered an appropriate measure to achieve complete cytoreduction after the patient had completed childbearing. The recommended completion surgery was performed in January 2022, and histopathological examination showed no residual tumor cells in the excised tissue.

Various treatment options were discussed at the following tumour conference, although it should be pointed out once again that there is no clear evidence for a superior treatment option given the weak data available: (1) close observation, (2) repeat chemotherapy (e.g., carboplatin/paclitaxel or cisplatin/etoposide/ifosfamide (PEI)), (3) sole antihormonal therapy, or (4) a combination of chemotherapy and antihormonal therapy. In the end, the patient decided in favour of starting anti-hormonal therapy with letrozole after considering all the options.

During the latest follow-up in November 2025, the patient showed no tumor-specific symptoms. She is still under treatment with Letrozole. Furthermore, the bone density measurements appear to be unremarkable and tumor markers are in the norm. The patient undergoes regular follow-ups, where therapeutic options are being continuously re-evaluated. Given the rarity of recurrence during pregnancy and the diagnostic challenges encountered in this case, the following section discusses the biological and clinical considerations surrounding pregnancy after aGCT.

**Figure:**
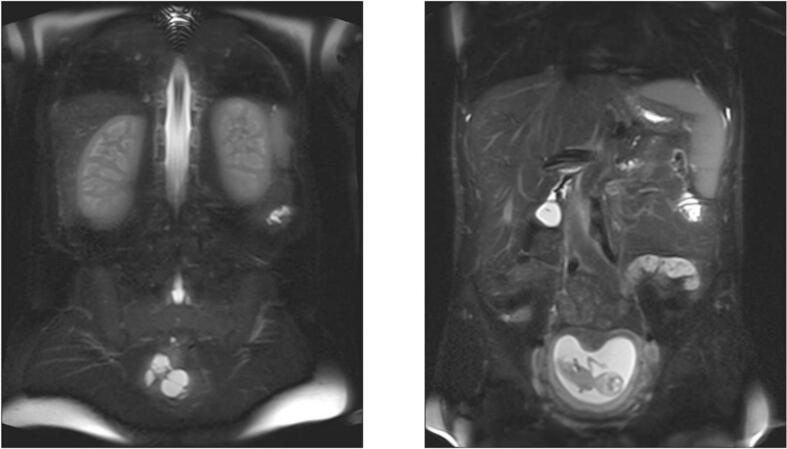

II) July 2018, Abdomen and pelvis, Coronary slices (frontal plane), Sequence: T2-weighted MRI sequence, no contrast-enhancement.

**Figure:**
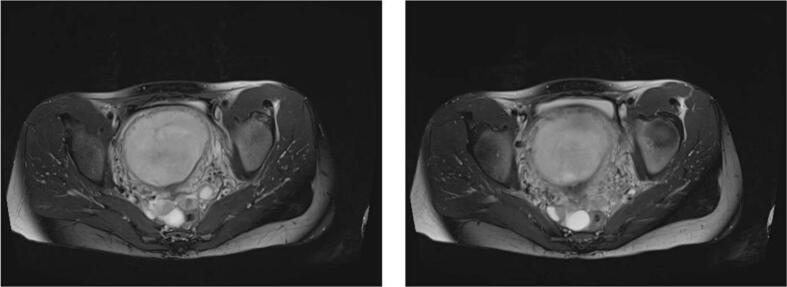

III) July 2018, Small pelvis (Pelvis minor), Axial (transversal sections), Sequence: T2-weighted sequence, No contrast agent administration.

## 3 Risks of pregnancy after aGCT, discussion

The question of the compatibility of pregnancy and a history of adult granulosa cell tumour poses a particular clinical challenge, especially due to the potential influence of hormonal changes on tumour behaviour. There are several pathophysiological and clinical arguments in favour of proceeding with caution when planning a family after an aGCT.

One central aspect is the hormone-dependent growth potential of aGCT. These tumours belong to the sex cord-stromal tumours of the ovary and are typically hormone-active − in around 70% of cases they produce oestrogens (Schumer and Cannistra, 2003). In addition, many GCT show expression of receptors for oestrogen, progesterone and follicle-stimulating hormone (FSH) (Jamieson and Fuller, 2012). As the neoplastic cells are degenerated granulosa cells that proliferate in an FSH-dependent manner during normal ovarian function, a stimulating effect through hormonal changes − as they occur during pregnancy − appears biologically plausible (Jamieson and Fuller, 2012).

During pregnancy, serum concentrations of estrogens, progesterone, and human chorionic gonadotropin (hCG) rise markedly, creating a hormonal milieu that may theoretically promote the proliferation of residual tumor cells (Jamieson and Fuller, 2012). This suspicion is supported by isolated case reports in which tumour growth or recurrence was observed during or immediately after pregnancy (Zanagnolo et al., 2004).

Another practical aspect concerns the difficulty of diagnosis and tumour monitoring during pregnancy. Routine surveillance is further complicated by physiological changes in pregnancy, which may limit the interpretability of tumor markers such as inhibin B. Imaging procedures are also limited, as CT and contrast-enhanced MRI should be avoided during pregnancy, which restricts the diagnosis of suspected recurrence.

Although the interpretation of inhibin B during pregnancy is limited, a marked and dynamic increase outside the expected physiological range was observed in this case. Rather than relying on absolute values, marker kinetics were considered in conjunction with imaging findings and clinical context. This approach supported the suspicion of tumor recurrence, which was subsequently confirmed intraoperatively. Inhibin A was not routinely assessed, as inhibin B represents the primary tumor marker used for disease monitoring in adult granulosa cell tumors (Schumer and Cannistra, 2003).

## 4 Counterarguments: Why a general recommendation against pregnancy is not justified

Despite the potential risks that pregnancy after a granulosa cell tumour (GCT) can entail, there are equally weighty arguments against a general ban or a fundamental rejection of pregnancy in affected patients. These relate to the limited evidence base, clinical experience with complication-free pregnancies after GCT as well as the psychosocial and reproductive aspects in mostly young patients.

A decisive argument against a general ban is the lack of reliable prospective data proving that pregnancy significantly increases the risk of recurrence (Ray-Coquard et al., 2014). The current data situation is based almost exclusively on case reports and retrospective analyses, which at best provide indications of a possible association, but cannot prove a causal relationship (Ray-Coquard et al., 2014). Several reports on patients with a history of GCT have documented complication-free pregnancies without tumour progression (Zanagnolo et al., 2004, Ayhan et al., 2005). Late recurrences are a known characteristic of GCT, often only occurring after several years − regardless of whether a pregnancy was present or not (Schumer and Cannistra, 2003). This suggests that a possible reactivation is not exclusively hormonally triggered, but has multifactorial causes. Therefore, a ban based solely on a pregnancy-induced tumour recurrence does not appear to be scientifically sound.

Another important aspect is the fertility issue itself: Many patients with GCT are premenopausal, and it is not uncommon for fertility-preserving surgery to be performed (Mangili et al., 2013). A later pregnancy is a central life wish for these patients and is a relevant part of coping with the disease. Studies show that losing the ability to reproduce can be a considerable psychological burden (Carter et al., 2010). A general ban on pregnancy could therefore cause more harm than good − especially if a patient has already been tumour-free for years.

In addition, the rare but benign pregnancy luteoma must be considered as a differential diagnosis during tumor monitoring in pregnancy (Clement and Young, 1988). Due to its imaging appearance and laboratory findings—particularly elevated inhibin B levels—it can closely resemble a GCT (Young, 2005). This presents a diagnostic challenge, especially in patients with a desire to preserve fertility, and should be taken into account to avoid unnecessary surgical interventions.

## 5 Conclusion

The question of whether pregnancy after a GCT is oncologically justifiable cannot be answered in general terms, but requires a differentiated, patient-specific approach. Biologically and pathophysiologically, there are several arguments in favour of a potential growth-promoting effect of pregnancy, in particular due to the hormonal changes associated with a massive increase in oestrogens, progesterone and hCG (Schumer and Cannistra, 2003, Jamieson and Fuller, 2012).

At the same time, it must be emphasised that there are no reliable prospective studies that identify pregnancy as an independent risk factor for recurrence after GCT (Ray-Coquard et al., 2014). Numerous clinical observations show complication-free pregnancies in patients with a history of GCT (Zanagnolo et al., 2004, Ayhan et al., 2005). A general ban on pregnancy would also contradict the desire of many young patients to fulfil their wish to have children and could significantly impair psychosocial disease management (Carter et al., 2010).

Current international consensus recommendations consider a fertility-sparing treatment approach to be oncologically acceptable in young patients with early-stage adult granulosa cell tumors, provided that careful intraoperative assessment and adequate staging are performed (Ray-Coquard et al., 2014). This approach is supported by case reports and small series describing successful pregnancies following or during GCT disease, emphasizing the importance of individualized patient assessment (Zanagnolo et al., 2004, Ayhan et al., 2005).

Against this background, it seems reasonable not to generally rule out pregnancies after GCT, but to allow them under certain conditions: The prerequisite could be a tumour-free follow-up period, ideally of at least two years, as well as regular monitoring of tumor markers such as inhibin B, and targeted imaging—primarily transvaginal ultrasound—with additional cross-sectional imaging if recurrence is suspected.

Written informed consent was obtained from the patient for publication of this case report and any accompanying images.

**Ethics statement:**

Informed consent was obtained from the patient for publication of this case report.

**Declaration of generative AI and AI-assisted technologies in the manuscript preparation process**

During the preparation of this work the author used ChatGPT in order to assist with phrasing, organization, and refinement of the text. After using this tool, the author reviewed and edited the content as needed and takes full responsibility for the content of the published article.

## CRediT authorship contribution statement

**Moritz Matthaei:** Writing – original draft. **B. Gebauer:** . **A. Kunze:** . **W. Schmitt:** . **D. Dimitrova:** . **J. Sehouli:** .

## Declaration of competing interest

The authors declare that they have no known competing financial interests or personal relationships that could have appeared to influence the work reported in this paper.
